# Regulation of genes related to immune signaling and detoxification in *Apis mellifera* by an inhibitor of histone deacetylation

**DOI:** 10.1038/srep41255

**Published:** 2017-01-23

**Authors:** Yee-Tung Hu, Tsai-Chin Wu, En-Cheng Yang, Pei-Chi Wu, Po-Tse Lin, Yueh-Lung Wu

**Affiliations:** 1 Department of Entomology, National Taiwan University, Taipei 106, Taiwan

## Abstract

The western honeybee (*Apis mellifera*) is essential for the global economy due to its important role in ecosystems and agriculture as a pollinator of numerous flowering plants and crops. Pesticide abuse has greatly impacted honeybees and caused tremendous loss of honeybee colonies worldwide. The reasons for colony loss remain unclear, but involvement of pesticides and pathogen-pesticide interactions has been hypothesized. Histone deacetylase inhibitors (HDACis) inhibit the activity of histone acetylase, which causes the hyperacetylation of histone cores and influences gene expression. In this study, sodium butyrate, an HDACi, was used as a dietary supplement for honeybees; after treatment, gene expression profiles were analyzed using quantitative PCR. The results showed that sodium butyrate up-regulated genes involved in anti-pathogen and detoxification pathways. The bioassay results showed that honeybees treated with sodium butyrate were more tolerant to imidacloprid. Additionally, sodium butyrate strengthened the immune response of honeybees to invasions of *Nosema ceranae* and viral infections. We also performed a bioassay in which honeybees were exposed to pesticides and pathogens. Our results provide additional data regarding the mechanism by which honeybees react to stress and the potential application of HDACis in beekeeping.

*Apis mellifera*, also known as the western honeybee, belongs to the order Hymenoptera and the family Apidae. Western honeybees are vital economic resources because they pollinate most flowering plants^1^. In recent years, an increasing number of colonies worldwide have been affected by missing worker bees, a condition termed colony collapse disorder (CCD). CCD has caused significant economic losses. This mysterious phenomenon may be caused by pathogens^2^, pesticides^3^, or even interactions between those two factors by creating stressful environments for honeybees^4^.

Social insects such as honeybees have fewer immune-related genes, i.e., they have weaker defenses against pathogens^5^. Honeybees are susceptible to infection by viruses that might cause colony diseases, such as chronic bee paralysis virus (CBPV) and acute bee paralysis virus (ABPV)^6^. Pesticides are another key factor in CCD. Chemical pesticides are considered the most time- and cost-effective method for pest management. Approximately 120 types of pesticides with varying effects on bees have been detected in beehives^7^. Certain pesticides interfere with insect neurophysiology, and others may affect insect development, adult longevity, immunology, and fecundity^8^,^9^. Interactions between pathogens and pesticides have a synergistic effect on bees, as observed in the interaction between *Nosema* spp. and pesticides^10^,^11^. Honeybees administered imidacloprid, a neurotoxin that can affect behavior, exhibit dose-dependent changes in their locomotor activity^12^. Other studies have concluded that imidacloprid and other neonicotinoid insecticides influence olfaction learning and interrupt orientation and navigation^13^.

In eukaryotic cells, DNA sequences are packed with histone cores, which are composed of several histone subunits: H2A, H2B, H3 and H4. Each subunit contains amino-acid tails that are sites of post-translational regulation^14^. Histone deacetylases (HDACs) modify chromatin structures by removing acetyl from histone tails at specific lysine sites and play an important role in epigenetic gene regulation^15^. DNA methylation and histone modification are two types of major epigenetic modification^16^. Histone modifications include methylation of lysine and arginine, phosphorylation of serine, ubiquitination of lysine, and acetylation of lysine^17^. Different patterns or types of histone modification may up- or down-regulate gene expression^18^.

Two classes of enzymes control the acetylation status of histones: histone acetyltransferases and histone deacetylases. The functions of these two types of enzymes result in opposing gene expression outcomes^16^. Histone deacetylase inhibitors (HDACis) trigger histone tail acetylation, which leads to gene activation and can cause changes in gene expression of approximately 2–10%, depending on the cancer cell line^17^,^19^. Epigenetic modification can be triggered by environmental factors such as heavy metals or persistent organic pollutants, which can modulate epigenetic marks such as acetylation or methylation^20^. HDACis can accelerate growth, extend longevity and help overcome injuries in insects^21^,^22^. However, a high dose may arrest cell growth and induce apoptosis^23^,^24^. There have been several studies of to the effects of HDACis in insects^25^,^26^,^27^. Here, we sought to examine the effects of an HDACi on gene expression in insects. Sodium butyrate targets HDAC class 1 and 2a and can selectively modify all nucleosomal histones^28^,^29^. Butyrate is a short-chain fatty acid with deacetylase-inhibition activities that can alter gene expression in humans and mice^30^. A limited study of HDACis and honeybees used HDACis to study epigenetic modifications in honeybee workers and queens, as well as development^31^.

In this study, we specifically assessed the gene-expression profiles of honeybees altered by an HDACi (sodium butyrate) using PCR array. A total of 77 genes involved in immunity and detoxification were investigated. Sodium butyrate slightly up-regulated the immune-related genes of honeybees. Likewise, sodium butyrate up-regulated most detoxification genes. Interestingly, butyrate had a synergistic effect with imidacloprid in inducing resistance expression. Bioassays were used to evaluate the effect of sodium butyrate on honeybees exposed to imidacloprid or viral infections. Our results suggest that sodium butyrate enhances gene expression to defend honeybees against stress. Elucidating the regulation of genes by sodium butyrate may provide additional data regarding the mechanisms used by honeybees under adverse conditions.

## Results

### Effects of sodium butyrate on immunity gene signaling factors and anti-microbial peptides

**Figure 1:**
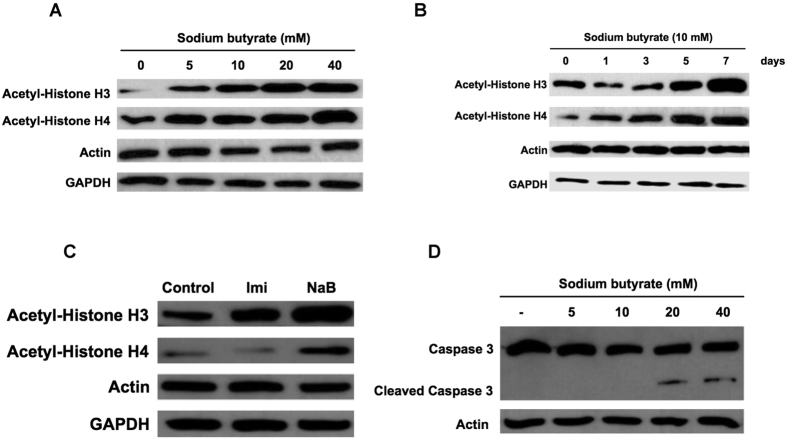
Regulation of acetyl-histone and caspase-3 expression at different concentrations of sodium butyrate and imidacloprid. (**A**) Western blot analysis of acetyl-H3 and acetyl-H4 expression in honeybee after 7 days of sodium butyrate treatment at different doses. The expression of actin and GAPDH was detected as a loading control. (**B**) Western blot analysis of acetyl-H3 and acetyl-H4 expression in the feeding assay for 1, 3, 5 and 7 days with 10 mM sodium butyrate. Expression of actin and GAPDH was used as the loading control. (**C**) Western blot of acetyl-H3 and acetyl-H4 in the feeding assay for sodium butyrate and imidacloprid with actin and GAPDH as the loading control. Imi, imidacloprid treatment; NaB, sodium butyrate treatment. (**D**) Western blot analysis of caspase-3 expression with and without sodium butyrate pretreatment. The caspase-3 and cleaved caspase-3 were detected by western blot. Expression of actin was used as the loading control.

Sodium butyrate is an HDACi and induces acetylation of the histone core^32^. In this study, we examined histone acetylation in response to sodium butyrate exposure in nurse bees using western blotting. Sodium butyrate concentrations of 5 mM, 10 mM, 20 mM and 40 mM (Fig. 1A) dissolved in ddH_2_O were used in the feeding assay for 1, 3, 5 and 7 days to identify the proper dose (Fig. 1B). Decreased expression of acetyl-H3 and acetyl-H4 in early time points has been occasionally observed. This may be due to physiological variation in individual bees collected for this experiment. Nevertheless, steady increase in the expression of both proteins was consistently detected in all experiments, which correlated with increase in gene expression after day 5 of sodium butyrate treatment. Western blot analysis showed that the level of histone acetylation (acetyl-H3 and acetyl-H4) significantly increased with addition of sodium butyrate and this increase was dose dependent. We also compared sodium butyrate with imidacloprid on the effect on histone modification. Western blot showed histone acetylation enhancement in sodium butyrate treated bees, but not in the imidacloprid treated group. These finding support our hypothesis that sodium butyrate induces histone modification and therefore enhances gene expression (Fig. 1C). Induction of apoptosis has been previously observed at high doses of sodium butyrate^33^,^34^, and we therefore assessed the induction of apoptosis in bees in response to different concentrations of sodium butyrate. As expected, the caspase-3 was not processed to its active subunit in low concentrations (5 mM and 10 mM). Yet, at higher concentrations (20 mM and 40 mM), it is proven to be processed to the active subunit (Fig. 1D). Based on this result, we exposed nurse bees to 10 mM sodium butyrate for 7 days to induce the expression of acetyl-H3 and acetyl-H4 but not caspase-3. We focused on the expression of immune and detoxification genes using a PCR array.

**Figure 2:**
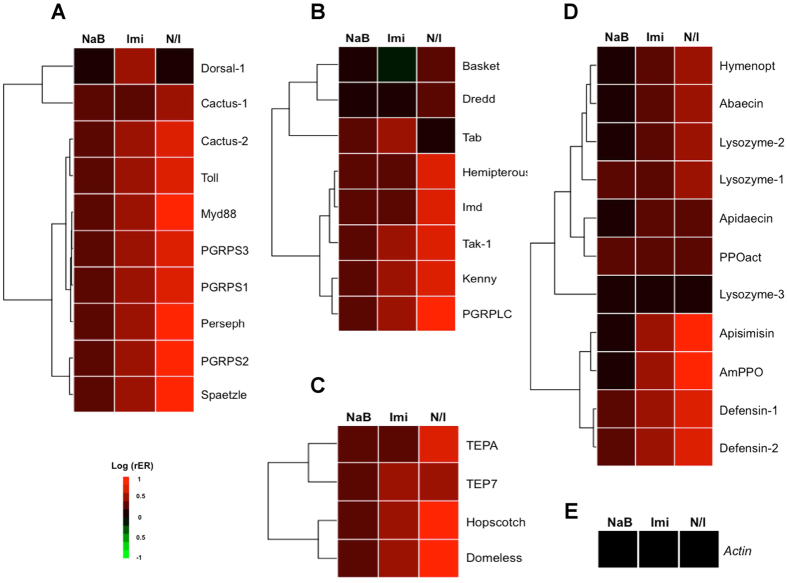
Relative expression (rER) of immune-related genes. (**A**) Toll pathway, (**B**) Imd/JNK pathway, (**C**) JAK/STAT pathway, and (**D**) anti-microbial peptide. (**E**) Expression of actin was used as the control. The scale is the logarithm of the relative fold change (Control group = 1). NaB, sodium butyrate; Imi, imidacloprid; N/I, sodium butyrate/imidacloprid treatment. Clustering analysis was based on the Euclidean distance.

Pesticides have adverse effects on the bee immune system^35^. Several studies have revealed that neonicotinoid pesticides such as imidacloprid induce pathogen outbreaks in honeybees^10^,^36^. Nurse bees were treated with sodium butyrate, imidacloprid or both chemicals for 24 h to determine the influences of these chemicals on the immune system. We used quantitative reverse transcription PCR (RT-qPCR) to monitor the expression of immune pathways (Fig. 2A,B and C) and anti-microbial peptides (AMP)(Fig. 2D), including 33 immune-related genes from four pathways (Toll, Imd, JNK and JAK/STAT), among the three test groups (sodium butyrate, imidacloprid, and sodium butyrate/imidacloprid). In the imidacloprid treatment group, genes with relative expression levels more than 3-fold higher than that in the control group were selected for further discussion. In the co-treatment group (sodium butyrate/imidacloprid), statistically significant differences in gene expression levels that were two-fold higher than those in the imidacloprid group might be related to a synergistic effect between imidacloprid and sodium butyrate (Table 1). The sodium butyrate/imidacloprid treatment exhibited the highest levels of immune-related gene expression (Fig. 2). This outcome indicates that sodium butyrate and imidacloprid increased gene expression and had a synergistic effect.

**Figure 3:**
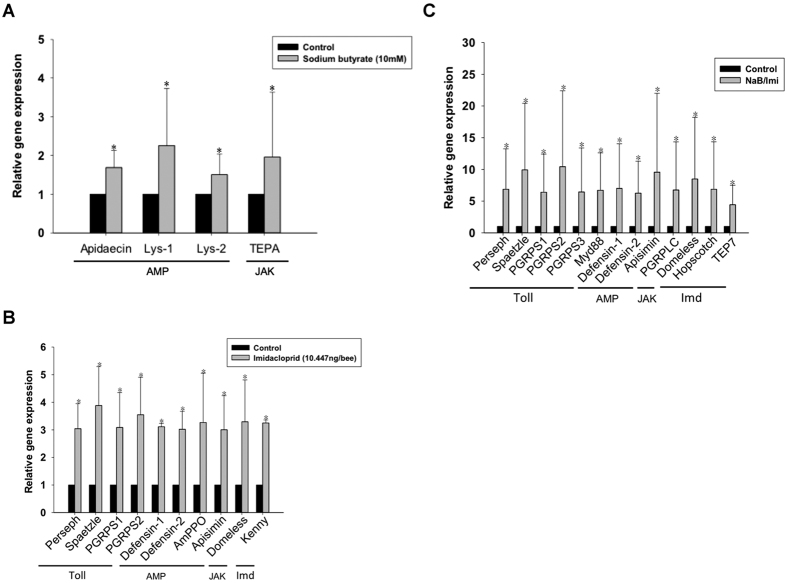
Relative expression of immune-related genes with significant changes (*p* < 0.05). (**A**) Sodium butyrate treatment, (**B**) imidacloprid treatment, (**C**) Sodium butyrate (NaB)/imidacloprid (lmi) treatment. The black bar represents the control group; the grey bar represents the treatment group. Toll, Toll pathway; AMP, anti-microbial peptide; JAK, JAK/STAT pathway; Imd, Imd/JNK pathway. The values from the control groups were set to 1. The fold changes were compared to those in the control groups. All results were analyzed based on data collected from three independent experiments and assessed by the Mann-Whitney *U*-test.

Sodium butyrate up-regulated the expression of apidaecin, lysozyme-1, lysozyme-2 and thioester-containing proteins A (TEPA) from the JAK/STAT pathway (*p* < 0.05) to levels slightly higher than those in the control group (Fig. 3A). Imidacloprid induced the expression of more genes than sodium butyrate alone, including upstream Toll-signaling molecules such as *PGRPS1, PGRPS2, persephone*, and *spaetzle* in the Toll pathway, *domeless* in the JAK/STAT pathway and *kenny* in the Imd/JNK pathway. The expression of four AMP genes (*defensin-1, defensin-2, AmPPO* and *apisimin*) was induced by imidacloprid (Fig. 3B). Treatment with sodium butyrate and imidacloprid together induced higher expression of more genes than treatment with sodium butyrate or imidacloprid alone. Sodium butyrate and imidacloprid induced the expression of more types of anti-microbial peptides and higher levels of expression compared to either treatment alone. These factors may indicate a strong immune response (Figs 2D and 3C).

### Effects of sodium butyrate on the expression of detoxification genes

**Figure 4:**
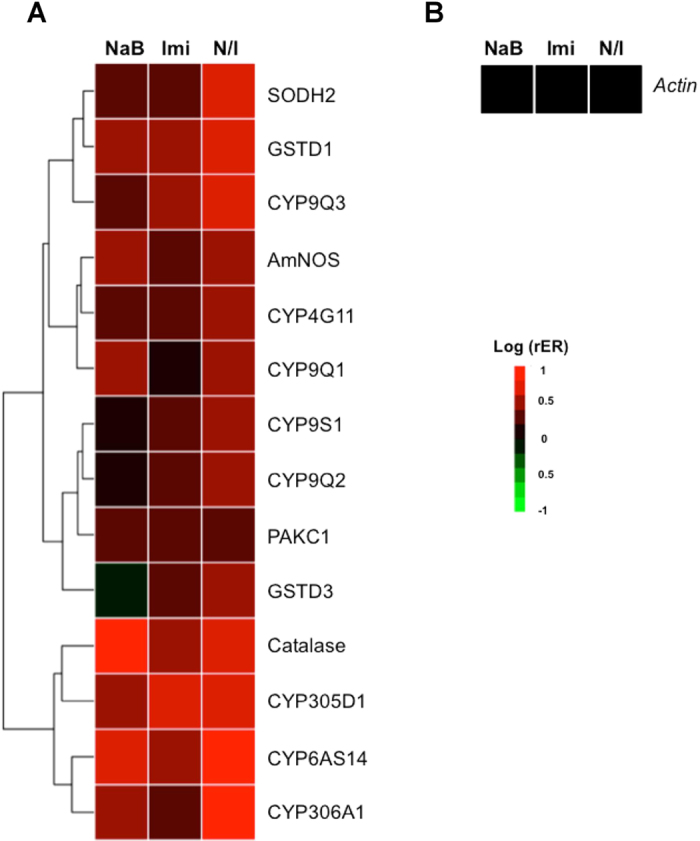
Relative expression (rER) of detoxification genes. (**A**) The scale was the logarithm of the fold change (control group = 1). (**B**) Expression of actin was used as the control. NaB, sodium butyrate; Imi, imidacloprid; N/I, sodium butyrate/imidacloprid treatment. Clustering analysis was based on the Euclidean distance.

**Figure 5:**
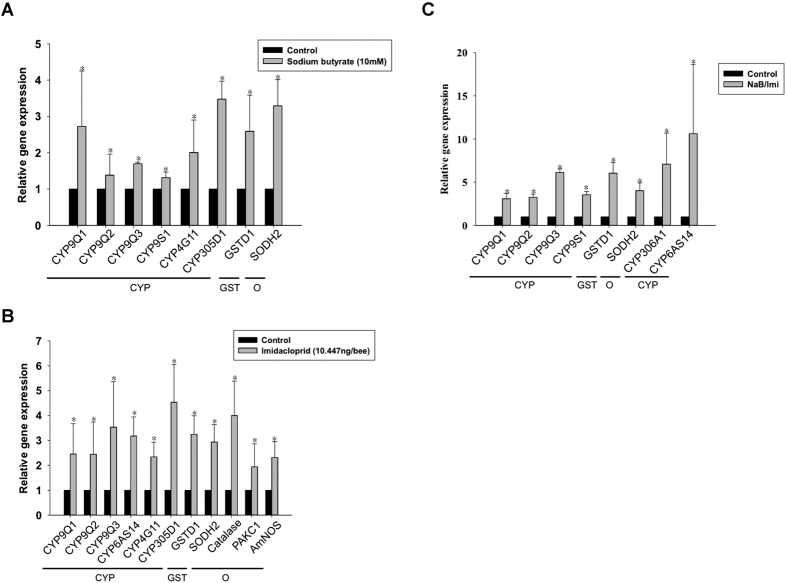
Relative expression of detoxification genes with significant changes (*p* < 0.05). (**A**) Sodium butyrate treatment, (**B**) imidacloprid treatment, and (**C**) sodium butyrate (NaB)/imidacloprid (Imi) treatment. The black bar represents the control group; the grey bar represents the treatment group. CYP, Cytochrome p450 monooxygenases (P450s); GST, Glutathione-S-transferases (GST); O, Other. The results from the control groups were set to 1. The fold changes were compared to the data in the control groups. All experiments were performed with at least three replicates, and the data were assessed by the Mann-Whitney *U*-test.

In insects, cytochrome P450 (CYP gene), glutathione-S-transferase (GST) and other oxidative-stress enzymes are responsible for pesticide resistance. Because sodium butyrate exhibited positive effects on the immune system of nurse bees, we further explored the influence of sodium butyrate on the expression of detoxification genes. Fourteen detoxification-related genes were studied. Genes with higher relative gene expression in the co-treatment group than the groups treated with sodium butyrate or imidacloprid alone were selected for further study. The expression patterns of the genes that responded significantly are presented in Fig. 4 and Table 2. Treatment with sodium butyrate for 7 days enhanced the expression of a number of genes that are related to pesticide responses, such as those in the CYP9 family, CYP4G11, superoxide dismutase (SOD), P450s and GSTs. The CYP9 and CYP4G11 families are responsible for the synthesis of detoxification enzymes for neonicotinoid pesticides (Fig. 5A). Co-treatment with sodium butyrate and imidacloprid induced the expression of more genes than the individual treatments and the highest levels of expression (Fig. 5C). SOD is involved in the detoxification of reactive oxygen species (ROS) and was up-regulated by these three treatments (Fig. 5A,B and C). In contrast to GSTD1, no differences in GSTD3 expression were observed among the treatments, which suggests that GSTD3 may not be involved in the detoxification of imidacloprid. There was a strong positive correlation between the effects of the sodium butyrate and imidacloprid treatments on most detoxification P450 genes. Therefore, we propose that sodium butyrate may contribute to pesticide resistance in honeybees.

### Sodium butyrate confers imidacloprid resistance to both forager and nurse bees

As described above, sodium butyrate induced a higher level of expression of detoxification genes when nurse bees were also treated with imidacloprid. We further determined the LD_50_ in honeybees treated with different doses of sodium butyrate and imidacloprid together and with sodium butyrate alone. A total of 30 forager or nurse bees were collected for the estimation of LD_50._ The bees received sodium butyrate for 7 days, followed by imidacloprid for 15 days and no treatment for 15 days. In forager bees, an imidacloprid dose of 64.649 ng/bee resulted in the death of approximately 70% without sodium butyrate treatment. A dose of 10.447 ng/bee of imidacloprid dissolved in acetone solution was used to feed nurse bees and killed approximately 80% (Table 3). Thus, forager bees are more tolerant to imidacloprid than nurse bees (Table 3), possibly reflecting the exposure of forager bees to the stressful wild environment. This observation is consistent with previous studies that suggested that the resistance of honeybees increases as they age^37^.

**Figure 6:**
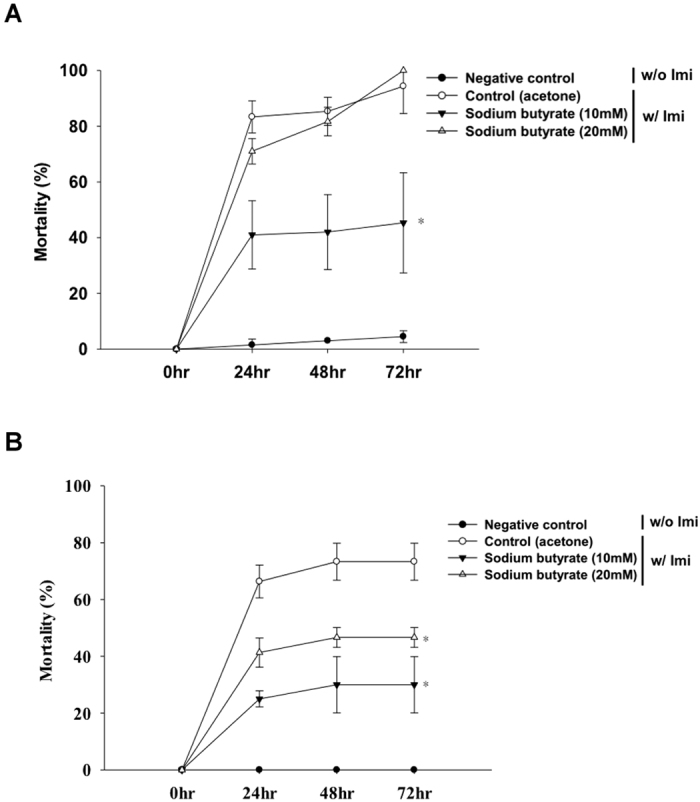
Mortality of (**A**) forager and (**B**) nurse bees treated with imidacloprid. The black triangle represents bees treated with 10 mM sodium butyrate and imidacloprid (forager bees, 64.649 ng/bee; nurse bees, 10.447 ng/bee); the white triangle represents bees treated with 20 mM sodium butyrate and imidacloprid (forager bees, 64.649 ng/bee; nurse bees, 10.447 ng/bee). The black circle represents bees not treated with sodium butyrate and imidacloprid (H_2_O only); the white circle represents bees treated with solvent (acetone) and imidacloprid. The data are presented as the mean ± standard deviation. Statistical analysis was performed using the Mann-Whitney *U*-test, *p < 0.05 relative to data collected from the group treated with solvent alone. Imi, imidacloprid.

Forager bees that consumed a sucrose solution mixed with 10 mM sodium butyrate and imidacloprid exhibited a mortality of 40%, whereas no difference in mortality was observed between bees treated with 20 mM sodium butyrate and the control group (Fig. 6A). The mortality of nurse bees was approximately 30% in the sodium butyrate (10 mM) and imidacloprid treatment group and 40% in the 20 mM sodium butyrate with imidacloprid group (Fig. 6B). This result indicates that sodium butyrate protects honeybees against pesticides but might also have toxicity above a certain dose.

### Counting *Nosema ceranae* spores and honeybee virus infection

**Figure 7:**
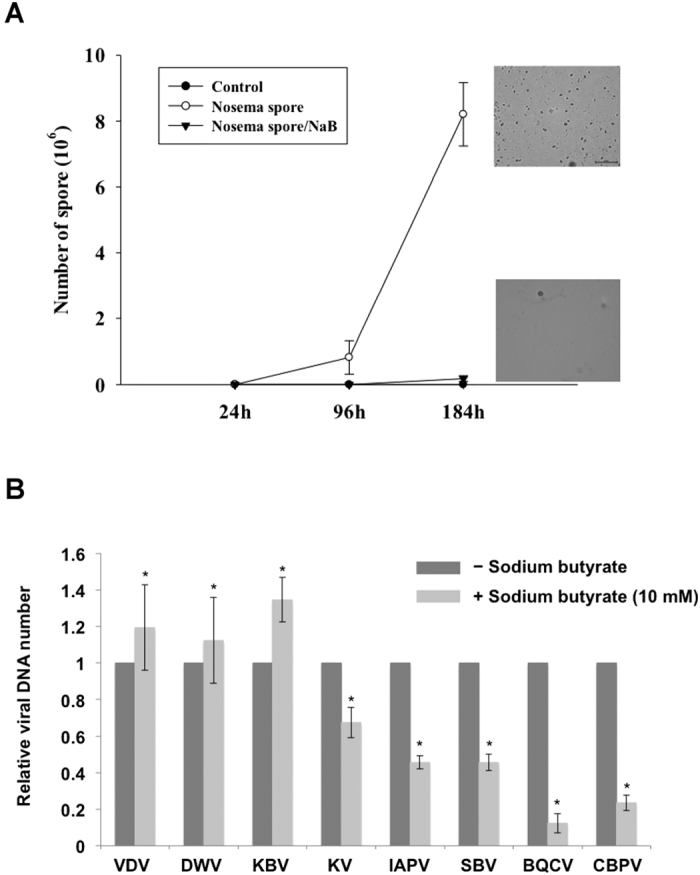
Sodium butyrate suppresses pathogen activity in infected honeybees. (**A**) The number of *Nosema* spores in the honeybee mid-gut during the infection period. The honeybees were infected with 1 × 10^5^ spores. The white circle represents bees infected with *Nosema* spores but not treated with sodium butyrate; the black triangle represents bees infected with *Nosema* spores and treated with sodium butyrate (10 mM); and the black circle represents bees without *Nosema* infection and treated with sodium butyrate (N = 3). (**B**) Analysis of viral DNA in infected honeybees. Relative viral DNA replication was analyzed by RT-qPCR. VDV, *Varroa destructor* virus; DWV, deformed wing virus; KBV, Kashmir bee virus; KV, Kakugo virus; IAPV, Israel acute paralysis virus; SBV, sacbrood virus; BQCV, black queen cell virus; CBPV, chronic bee paralysis virus. The data are presented as the mean ± standard deviation. The values from the untreated groups were set to 1. Statistical analysis was performed using the Mann-Whitney *U*-test, *p < 0.05 relative to data collected from the control group.

As previously described, several AMP genes were up-regulated by exposure to sodium butyrate. We used sodium butyrate to treat fungus- and virus-infected bees. *N. ceranae* is a fungal pathogen that inhabits the mid-guts of honeybees and suppresses the honeybee immune system to facilitate spore proliferation. In this study, bees treated with sodium butyrate were challenged to determine if sodium butyrate can enhance the immune response and protect bees against fungal infections. Bees were separated into two groups after artificial infection with 1 × 10^5^ spores by oral feeding. One group was fed a regular sucrose solution as a control; the other was fed a sucrose and sodium butyrate solution to assess the effect of sodium butyrate on immune stimulation. The number of spores was determined on days 1, 4 and 7 by dissecting the mid-gut and counting with a hemocytometer. The bioassay showed that *Nosema* spores were minimal in both groups on day 4 and significantly differed on day 7 (Fig. 7A). A high concentration of spores was observed in the mid-gut of bees not treated with sodium butyrate treatment on day 7. By contrast, a lower number of spores was observed in the sodium butyrate group. Thus, we propose that sodium butyrate may help honeybees overcome *Nosema*-mediated immune suppression and further inhibit the growth of spores.

More than 8 persistent infectious viruses are common among western bees in Taiwan. We treated infected honeybees with sodium butyrate to explore the effect of sodium butyrate on suppressing viral activity in the hosts. RT-qPCR showed a significant decrease in viral DNA expression in sodium butyrate-treated bees, except for KBV virus (Fig. 7B). KBV viral expression was unresponsive to sodium butyrate, which implies that the KBV infection might not induce an immune response in honeybees. In the *Nosema* and viral DNA-expression tests, sodium butyrate suppressed pathogen activities in infected honeybees. This finding indicates that sodium butyrate can induce the expression of immune and detoxification genes in honeybees, resulting in a reduced pathogen copy number and mortality rate in bees.

## Discussion

Our study evaluated the influence of an HDACi and pesticides on the immune system and detoxification in honeybees. This study of gene expression and gene interaction with sodium butyrate and imidacloprid may shed light on how honeybees cope with external stress. Although this system is well studied in mammals, the mechanisms in insects remain unknown. Our bioassays provide valuable information on HDACi gene regulation at the epigenetic level. Previous studies on the immune response and detoxification are reviewed and discussed below to reveal specific defensive mechanisms against microorganisms and insecticides.

Sodium butyrate is a short-chain fatty acid molecule that targets class 1 and 2a HDACs. The pathways by which HDACs and HDACis regulate immunity in mammals have been determined^32^,^38^,^39^, but only a limited number of studies have been performed on insect immunity and detoxification. Previous studies have demonstrated that high sodium butyrate concentrations induce apoptosis in targeted cells^33^,^34^. We observed high sodium butyrate concentrations induced apoptosis in bees, whereas low sodium butyrate concentrations did not (Fig. 1D). To avoid induction of apoptosis by sodium butyrate treatment, which would also affect the expression of immunity-related genes and genes involved in detoxification, bees were treated with sodium butyrate concentrations that would result in the induction of acetyl-H3 and acetyl-H4 expression but not caspase-3 expression. Although the concentration of sodium butyrate was lower than that of imidacloprid, several immune-related genes were significantly induced by sodium butyrate. Three of four up-regulated genes were previously shown to be anti-pathogen peptides in insects (Fig. 2). Thioester-containing protein A (TEPA) is the end product of the JAK/STAT pathway. The transcriptional regulation of genes by HDACis by direct or indirect modulation is profound. Inhibition by HDACis may also be gene-specific, and the detailed mechanism remains to be further investigated. When treating bees with sodium butyrate and imidacloprid together, more immune-related genes were induced compared with treatment with imidacloprid or sodium butyrate alone. Most of the induced genes were the same as those induced in the imidacloprid group but with a higher expression level (Fig. 3C). This increase in expression may be the result of open chromatin, which caused a synergistic effect between sodium butyrate and imidacloprid.

This result also implies that most of the immune-related genes induced by imidacloprid are modified by HDAC1 and 2. In mammals, HDAC1 and 2 have a wide range of effects on the immune system^39^. HDAC1 and 2 bind to the NF-kB co-repressor and down-regulate NF-kB-mediated gene expression. In contrast to our results in the honeybee model, the inhibition of HDAC1 and 2 by sodium butyrate facilitates NF-kB proteins such as relish and dorsal to transcribe downstream AMP genes^40^. This result implies that insects have different pathways and mechanisms of HDAC and HDACi regulation of immune-related genes. Cactus is an IkB that binds to NF-kB to repress its activity in mammals. In fruit flies, cactus is involved in phagocytosis and anti-fungal peptide synthesis^41^,^42^. *N. ceranae* is a fungus that causes great losses of bees due to damage of epithelial cells in the mid-gut. A study revealed that *N. ceranae* suppresses several types of anti-microbial peptides^43^. Most insect AMPs are not sufficiently effective against fungal pathogens in the hemolymph; in the insect gut, the defensive mechanism is based on AMPs and ROS^44^. Sodium butyrate also stimulates the cellular immune response to eliminate fungal spores by encapsulation and phagocytosis. In our fungal challenge bioassay (Fig. 7A), we proposed that the lower mortality rate in sodium butyrate-treated bees was due to the inhibition of *Nosema* spore proliferation in the mid-gut because sodium butyrate stimulated the expression of AMPs. In the viral challenge, sodium butyrate also suppressed the viral expression level. This result indicates that sodium butyrate treatment can boost the immune response and protect honeybees from external stress from a variety of sources, including pesticides and pathogen infections (Figs 6 and 7).

Insecticides influence the immune system of insects in several ways, including both cellular and humoral immunity^42^,^45^. Neonicotinoid insecticides are a negative factor for honeybee immunity. Clothianidin down-regulates apidaecin, an anti-bacterial peptide from honeybee that is effective against a wide range of bacteria, after 6 h of bacterial infection^46^. In our study, we also focused on imidacloprid and its direct acute toxicity effects on honeybees without pathogen challenges after 24 h of treatment. In agreement with previous studies, imidacloprid induced several immune signaling genes and AMP genes that were previously reported to be significant anti-pathogen genes (Table 1). The immune-related genes *persephone* and *spaetzle* from the Toll pathway, *kenny* from the Imd pathway, *hopscotch* from the JAK/STAT pathway, P*GRPS1, PGRPS2, defensin 1*, and *defensin 2* were all induced by imidacloprid (Fig. 3B). Defensins are cysteine-containing peptides that target bacteria^45^. PGRPs also target pathogens and trigger an immune signaling pathway^47^. A study in which bee larvae were fed several pesticides mixed with sucrose until pupation demonstrated that imidacloprid induced *PPOact* and *PGRPs* in bee pupae^48^. In agreement with previous studies, our results showed that these immune-related genes (*persephone, spaetzle, kenny, hopscotch, PGRPS1, PGRPS2* and *defensins*) protect bees not only from pathogens but also from pesticides. The up-regulation of genes that were not reported to be anti-pathogenic (e.g., Toll and PGRPS3) by imidacloprid implies the alteration of gene expression in honeybees by pesticides and pathogens.

In the detoxification gene expression profiling, the effect of sodium butyrate on detoxification genes was similar to that of imidacloprid (Fig. 5A and B), which suggests that most of the detoxification genes up-regulated by imidacloprid are modified by HDAC1 and 2. Sodium butyrate induces human CYP3A4 by 40-fold compared to untreated Caco-2 cells^49^. The detailed mechanism by which sodium butyrate interacts with cytochrome and increases P450 expression is not yet fully understood. The induction by sodium butyrate and the synergistic effects of sodium butyrate and imidacloprid are related to the inhibition of HDAC1 and 2. The bioassay results indicated that sodium butyrate protects honeybees against pesticides but may be toxic at higher doses (Fig. 6A and B).

For non-target insects such as honeybees, pesticides are deadly. Compared with *Drosophila melanogaster* and *Anopheles gambiae*, honeybees have lower cytochrome P450 and GST levels. Three subfamilies of P450, CYP4, 6 and 9, are the most common detoxification enzymes in other insects^50^,^51^. These differences among insects may explain the high sensitivity of honeybees to insecticides^52^. Honeybees also lack insect-specific Delta and Epsilon GSTs, which are two important classes of GSTs that regulate insecticide detoxification. In our study, imidacloprid induced the expression of P450s and GSTs, including CYP4, 6, 9 and *gstd1* (Fig. 5B), which may play a role in imidacloprid metabolism. In addition, two other genes induced by imidacloprid, *sod* and *catalase*, have been reported to be antioxidant enzymes that reduce ROS. ROS have immune importance and injure cells by damaging macromolecules^44^. Insecticides cause an overload of oxidative stress, thereby increasing the abundance of antioxidants^53^,^54^.

In conclusion, our study provides a new perspective on how epigenetics regulates different groups of genes in nurse bees. This study is the first to report how sodium butyrate affects a wide range of genes in insects, using the honeybee as a model. The effects of HDACi and its interaction mechanisms with target genes are sophisticated and involve a wide spectrum of biological processes. This study screened the influence of sodium butyrate and imidacloprid on genes related to the immune system and detoxification in honeybees. As a worldwide economic insect, the loss of honeybees has attracted much attention. However, much remains unknown regarding the causes, disrupted mechanisms, and potential treatment or prevention of CCD. Our group is joining the efforts to investigate the response of sodium butyrate-treated honeybees to pathogens or pesticides at the gene and bioassay levels. This assessment of the impacts of sodium butyrate treatment on the honeybee model offers insightful information to the community regarding the potential of HDACi use in beekeeping.

## Methods

### Bee rearing

Western honeybees (*Apis mellifera*) were collected from a bee farm in Taoyuan County, Taiwan. For the bioassay and gene analysis, experimental bees were divided into two groups: forager (for bioassay) and nurse bees (for bioassay and gene analysis). Foragers were collected outside the beehive, and nurse bees were collected from brood combs^55^; then, both groups were caged in a BugDorm (30 × 30 × 30 cm). Both groups of bees were kept in an incubator at 37 °C. The bees were fed a 50% sucrose solution (W/V) or formulated sucrose solution with different concentrations of sodium butyrate (5, 10, 20 and 40 mM/L; Tokyo Chemical Industry Co., Ltd.)^21^. A stock solution of 500 mM sodium butyrate in ddH_2_O was prepared. Nurse bees with eclosion on the same day were fed regular sucrose solution for a week to stabilize their physiological condition and then treated with a different sodium butyrate sucrose solution for another week. Forager bees were treated with sodium butyrate and regular food immediately for a week. At the end of the treatment, the bees were collected for gene analysis or bioassays.

### Western-blot analysis

A protein-extraction kit (Millipore) was used to extract honeybees proteins. Total protein was suspended in sample buffer (Bio-Rad). The samples were subjected to electrophoresis on SDS-PAGE gels with equal amounts of loaded protein. The proteins were transferred to nitrocellulose filters (Schleicher & Schuell) by electroblotting for 1 h in 200 mM glycine, 2.5 mM Tris/HCl, and 20% methanol. The filters were blocked with PBS containing 5% non-fat dried milk and 0.05% Tween-20 and incubated with primary antibodies against acetyl-H3, acetyl-H4, actin, GAPDH and caspase-3 (Millipore), followed by horseradish peroxidase-conjugated rabbit anti-mouse antibody (Millipore). The proteins were detected with an enhanced chemiluminescence system (Immobilon Western, Millipore).

### Total RNA preparation

Honeybee RNA was extracted using an RNA extraction kit (GeneMark). A total of four honeybees were pooled together for homogenization. The RNA was quantified using a NanoDrop 2000 spectrophotometer (Thermo Scientific).

### cDNA synthesis

cDNA synthesis was performed using a reverse-transcription kit (SuperScript^®^ III First-Strand Synthesis SuperMix). A total of 1 μg of RNA sample was used. The reaction was incubated in a PCR machine (Biometra) at 50 °C for 50 min and 85 °C for 5 min.

### Analysis of expression by RT-qPCR

The honeybees for gene analysis were partitioned into four groups: no treatment (acetone only), sodium butyrate only, imidacloprid only and sodium butyrate/imidacloprid treatment. The sodium butyrate and imidacloprid treatments were performed as described in the above steps. After imidacloprid treatment for 24 h, the RNA was extracted, and RT-qPCR was performed. For quantitative PCR, honeybee-specific gene primers for immunity (Table 4) and detoxification (Table 5) genes were used as described in previous studies^5^,^48^,^56^,^57^. Quantitative PCR was performed using an ABI PlusOne real-time system (StepOnePlus™, Applied Biosystems) with SYBR Green enzyme (BIOLINE). All samples were amplified simultaneously, and three independent experiments were performed. Raw Ct values are listed in Supplementary Tables S1 (immunity) and S2 (detoxification). GAPDH was included in each reaction as an internal standard, and relative gene expression was calculated using the 2^−ΔΔCt^ method.

### PCR-array images and data analysis

PCR-array images were analyzed with R statistics software. The fold change was calculated by the relative quantification method (2^−ΔΔCt^)^58^. Each group of tested genes was normalized to reference genes (GAPDH for immunity and detoxification genes); then, the fold change in the control group was used as a calibrator.

### Contact toxicity of imidacloprid

Imidacloprid commercial product (28.1%) was dissolved in 100% acetone, and 1 μL of the solution was dropped on the dorsal thorax of honeybees using a Hamilton PB-600 micro-applicator. Each treatment group included 30 bees. The dose of pesticide was based on the LD_50_, which was calculated with SPSS statistical software according to the mortality of self-rearing bees exposed to different dosages of imidacloprid (Table 3). The evaluation protocol was based on an EPA publication (EPA, 1995). The tested bees were caged in a plastic box (15 × 15 × 15 cm) in an incubator at 37 °C. The time course of the experiment was three days. The mortality was recorded each day. Each group included three replicates.

### Microsporidian infection and purification

The artificial infection and collection of *N. ceranae* spores was performed as described in a previous study^59^. The spore concentration was calculated with a hemocytometer. A group of 30 bees was treated with 1 × 10^5^ spores mixed with a 50% sucrose solution. Bees were held with forceps, and 10 μL of sucrose solution with 1 × 10^5^ spores was directly applied to the bee mouthpart using a Pipetman pipette. The tested bees were caged in a plastic box (15 × 15 × 15 cm) in an incubator at 37 °C. The bees in the cage were treated with 50% sucrose solution or sucrose with 10 mM sodium butyrate. The bees were collected at 1, 3 and 7 days to count the spores in the ventriculi. The ventriculi were dissected with forceps and homogenized with a plastic homogenizer in 20 μL of water. After homogenization, the plastic homogenizer was washed with another 20 μL of water to flush the remaining spores. The extract was filtered through cheesecloth, and the filtered liquid was centrifuged at 2,000 rpm for 30 min. The supernatant was inspected for suspended spores, which were removed. The pellet contained a high density of spores.

### Statistical analysis

The immunity and detoxification gene *C*t values from real-time PCR were normalized to the GAPDH *C*t values. The delta *C*t values were analyzed using the Mann-Whitney *U*-test for statistical significance using SPSS statistics software^60^. Statistical analysis of differences in target gene expression between two groups was performed using the Mann-Whitney *U*-test. A p-value < 0.05 indicated a statistically significant result.

## Additional Information

**How to cite this article**: Hu, Y.-T. *et al*. Regulation of genes related to immune signaling and detoxification in *Apis mellifera* by an inhibitor of histone deacetylation. *Sci. Rep.* **7**, 41255; doi: 10.1038/srep41255 (2017).

**Publisher's note:** Springer Nature remains neutral with regard to jurisdictional claims in published maps and institutional affiliations.

## Supplementary Material

Supplementary Table

## Figures and Tables

**Table 1:** Immune gene relative expression.

	Nab		Imidacloprid		NaB/Imi	
	Up	Dn	Up	Dn	Up	Dn
**Toll pathway**						
Perseph	1.88	—	3.04*	—	6.87*	—
Toll	1.71	—	2.98*	—	5.60*	—
Spetzle	2.08	—	3.80*	—	9.93*	—
PGRPS2	1.94	—	3.55*	—	10.44*	—
PGRPS3	1.8	—	2.90*	—	6.44*	—
Myd88	1.77	—	2.99*	—	6.71*	—
Cactus-1	1.66	—	2.27*	—	3.12	—
Cactus-2	1.89	—	2.79*	—	5.52*	—
Dorsal-1	1.6	—	2.69*	—	1.2	—
PGRPS1	1.78	—	3.09*	—	6.40*	—
**Imd/JNK pathway**						
Imd	1.79	—	2.30*	—	4.21*	—
Tak-1	1.66	—	2.67*	—	5.06*	—
Dredd	1.09	—	1.38*	—	2.13*	—
Kenny	1.8	—	3.25*	—	6.24*	—
Tab	1.91	—	2.14*	—	1.05	—
Hemipterous	1.92	—	2.44*	—	4.51*	—
Basket	1.54	—	0.92	—	1.6	—
PGRPLC	1.64	—	2.83	—	6.75*	—
**JAK/STAT pathway**						
TEPA	1.86*	—	2.19*	—	4.43*	—
Domeless	1.88	—	3.29*	—	8.49*	—
Hopscotch	1.63	—	2.85	—	6.87*	—
TEP7	2.04	—	2.68	—	3.01	—
**Antimicrobial peptides** (**AMP**)						
Abaectin	1.64	—	2.31*	—	4.18*	—
Defensin-1	1.75	—	3.11*	—	6.25*	—
Defensin-2	1.78	—	3.02*	—	7.00*	—
AmPPO	1.5	—	3.27*	—	11.5*	—
Apidaec	1.69*	—	2.62	—	2.38	—
Apisimin	1.63	—	3.01*	—	9.57*	—
Lys-1	2.26*	—	2.46	—	4.19*	—
Lys-2	1.51*	—	1.82*	—	3.56*	—
Lys-3	1.14	—	1.38	—	1.19	—
Hymenopt	1.32	—	2.41	—	4.41*	—

Asterisk means p-value < 0.05

**Table 2:** Detoxification gene relative expression.

	Nab		Imidacloprid		NaB/Imi	
	Up	Dn	Up	Dn	Up	Dn
**Cytochrome p450 monooxygenases** (**P450s**)						
CYP305D1	3.45*	—	4.53*	—	4.87*	—
CYP306A1	3.68	—	2.19	—	7.09*	—
CYP4G11	2.09*	—	2.34*	—	3.61*	—
CYP6AS14	4.23	—	3.18*	—	10.62*	—
CYP9Q1	2.75*	—	1.64*	—	3.08*	—
CYP9Q2	1.59*	—	2.45*	—	3.07*	—
CYP9Q3	1.70*	—	3.54*	—	6.12*	—
CYP9S1	1.54*	—	2.09	—	3.54*	—
**Glutathione-S-transferases** (**GST**)						
GSTD1	2.31*	—	3.25*	—	6.04*	—
GSTD3	0.89	—	1.66	—	3.62*	—
**Other**						
Catalse	7.68	—	4.00*	—	7.13*	—
PAKC1	1.72	—	1.95*	—	2.36*	—
AmNOS	3.19	—	2.32*	—	3.84*	—
SODH2	2.86*	—	2.94*	—	4.02*	—

Asterisk means p-value < 0.05

**Table 3:** Honeybee LD50 of imidacloprid in 24 hour.

Imidacloprid	Forager	Nurse
LD_50_ (ng/bee)	64.649	10.447

**Table 4:** Primer list of RT-qPCR genes (Immunity).

Gene name	Forward sequence	Reverse sequence
Persephone	CCGGTGAACTTGGAAAAGAT	ATCGCAATTTGTCCCAAAAC
Toll	TAGAGTGGCGCATTGTCAAG	ATCGCAATTTGTCCCAAAAC
Spaetzle	TGCACAAATTGTTTTTCCTGA	GTCGTCCATGAAATCGATCC
PGRPS1	TTTGAAAATTTCCTATGAAAGCA	TTTTTAATTGGTGGAGATGGAAA
PGRPS2	TAATTCATCATTCGGCGACA	TGTTTGTCCCATCCTCTTCC
PGRPS3	GAGGCTGGTACGACATTGGT	TTATAACCAGGTGCGTGTGC
PGRPLC	TCCGTCAGCCGTAGTTTTTC	CGTTTGTGCAAATCGAACAT
Myd88	TCACATCCAGATCCAACTGC	CAGCTGACGTTTGAGATTTTTG
Abaecin	CAGCATTCGCATACGTACCA	GACCAGGAAACGTTGGAAAC
Defensin-1	TGCGCTGCTAACTGTCTCAG	AATGGCACTTAACCGAAACG
Defensin-2	GCAACTACCGCCTTTACGTC	GGGTAACGTGCGACGTTTTA
Cactus-1	CACAAGATCTGGAGCAACGA	GCATTCTTGAAGGAGGAACG
Cactus-2	TTAGCAGGACAAACGGCTCT	CAGAAAGTGGTTCCGGTGTT
Dorsal-1	AAATGGTTCGCTCGTAGCAC	TCCATGATATGAGTGATGGAAA
PPOact	GTTTGGTCGACGGAAGAAAA	CCGTCGACTCGAAATCGTAT
AmPPO	AGATGGCATGCATTTGTTGA	CCACGCTCGTCTTCTTTAGG
Hymenopt	CTCTTCTGTGCCGTTGCATA	CGTCTCCTGTCATTCCATT
Apidaec	TAGTCGCGGTATTTGGGAAT	TTTCACGTGCTTCATATTCTTCA
Apisimin	TGAGCAAAATCGTTGCTGTC	AACGACATCCACGTTCGATT
Lys-1	GAACACACGGTTGGTCACTG	ATTTCCAACCATCGTTTTCG
Lys-2	CCAAATTAACAGCGCCAAGT	GCAATTCTTCACCCAACCAT
Lys-3	ATCTGTTTGCGGACCATTTC	TCGATGAATGCGAAGAAAATC
Imd	TGTTAACGACCGATGCAAAA	CATCGCTCTTTTCGGATGTT
Tak-1	ATGGATATGCTGCCAATGGT	TCGGATCGCATTCAACATAA
Dredd	GCGTCATAAAGAAAAAGGATCA	TTTCGGGTAATTGAGCAACG
Kenny	GCTGAACCAGAAAGCCACTT	TGCAAGTGATGATTGTTGGA
Tab	GCTATCATGCAGCTGTTCCA	ACACTGGGTCAGCCAATTTC
Hemipterous	CACCTGTTCAGGGTGGATCT	CCTTCGTGCAAAAGAAGGAG
Basket	AGGAGAACGTGGACATTTGG	AATCCGATGGAAACAGAACG
Domeless	TTGTGCTCCTGAAAATGCTG	AACCTCCAAATCGCTCTGTG
Hopscotch	ATTCATGGCATCGTGAACAA	CTGTGGTGGAGTTGTTGGTG
TEP7	GAGCCTACAGCCTCGTTTTG	CGGTTTCACGATTACGTCCT
TEPA	CAAGAAGAAACGTGCGTGAA	ATCGGGCAGTAAGGACATTG

**Table 5:** Primer list of RT-qPCR genes (Detoxification).

Gene name	Forward sequence	Reverse sequence
CYP9Q1	TCGAGAAGTTTTTCCACCG	CTCTTTCCTCCTCGATTG
CYP9Q2	GATTATCGCCTATTATTACTG	GTTCTCCTTCCCTCTGAT
CYP9Q3	GTTCCGGGAAAATGACTAC	GGTCAAAATGGTGGTGAC
CYP9S1	CTAATTTTCGCGTTCCCAAA	CTCCCGTTACGTTTGTCGAT
CYP6AS14	TGAAACTCATGACCGAGACG	AAAATTTGGGCCGCTAATAAA
CYP4G11	CAAAATGGTGTTCTCCTTACCG	ATGGCAACCCATCACTGC
CYP305D1	TCGATCTTTTTCTCGCTGGT	TTGCTTTGTCCTCCATGTTG
CYP306 A1	CGTCGATGGGAAGGATAAAA	TCGGTGAAATATCCCGATTC
GSTD1	GCCGCTTCAAAAGAAGTACG	GTGGCGAAAACAAGGATGAT
GSTD3	TGCATATGCTGGCATTGATT	TCCTCGCCAAGTATCTTGCT
SODH2	CAGTGCATGGTAGCCTGAGA	ACAGTGCTCCTTCAGCCAAT
Catalase	GTCTTGGCCCAAACAATCTG	CATTCTCTAGGCCCACCAAA
Am2446	CGCGCGAGTAAGAGAAAGAG	TCGAACAAGGGAAACGAAAC
PAKR1	GAAGCAATTATTCGGCAAGG	TCACCGAAACTTCCACCTTC
PAKC1	TCCATTTTTGGTCTCCTTGC	GTAAAAGCGCGAATGTGGTT
CEst01	TTTTGGGCCACGTTTACTTC	CAAATCGGTGGGTGTCTTCT
Am12900	TTAAGCAACCAACGCCTTTC	GGATCATGAAGCCACGAGAT
AmNOS	TCCACTCGCAGGTACTTTCC	TCTGGAGGATCACCATTTCC
Actin	TTCCCATCTATCGTCGGAAG	CTCTCTTTGATTGGGCTTCG
Aldoreductase	TAGTCCCCTTGGATCACCTG	TTGGGTCATCTGGTTTAGCC
Asn synthetase	TGGAATTTGGGCTCTTTTTG	TTCTGGACCACGGTGTGTAA
HAT P300	ACCAAGTGGAGGTCAACCTG	ATATTGTGGGTGGGCAAGAA
CREB	AATTGCAACCCAAGGTGAAG	TCAGTATGCACAAGGCCAAG
Cueball	CCAAAAGACGGGAAAAATGA	ACGCGTTAAAATCCCACTTG
DNMT1a	TGATCCAAAAACAGATGAGGAA	TACAGCACCATTCGGATGAC
DNMT1b	GAAATTACATGGGTGGGAGAA	GTCACTGCCTCTTCGAAACC
DNMT2	TGAGTCCTCCATGTCAACCTT	GCCAAATTGACAAGGGCTTA
DNMT3	CCTCCAACTGGACTTTGGAC	ACGTTCGGATTGTCCTTCAG
DWV	TCCATCAGGTTCTCCAATAACGGA	CCACCCAAATGCTAACTCTAAGCG
BQCV	TGGTCAGCTCCCACTACCTTAAAC	GCAACAAG AGAAACGTAAACCA
CBPV	GACCCCCGTTGGAACGACGC	CGGACGACGATTGGCGCTCA
SBV	GTGGCAGTGTCAGATAATCC	GTCAGAGAATGCGTAGTTCC
VDV	CCGTAGTTGGGAGATTGATG	GCGGGTACATCTTTCAGCTA
KV	GACTGAACCAAATCCGATGTC	GACTGAACCAAATCCGATGTC
IAPV	GCGGAGAATATAAGGCTCAG	CTTGCAAGATAAGAAAGGGGG
KBV	TATGCTGAACAACGCAAAGA	ACAACACGATGTCTGGGTTT
