# Supplementary material for: Regulation of genes related to immune signaling and detoxification in Apis mellifera by an inhibitor of histone deacetylation
Source: Sci Rep. 2017 Jan 23;7:41255. doi: 10.1038/srep41255 (PMC5253729; doi:10.1038/srep41255)
Supplement: Supplementary Table [file srep41255-s1.pdf]

Regulation of genes related to immune signaling and detoxification in *Apis mellifera* by an inhibitor of histone deacetylation

Yee-Tung Hu[1†], Tsai-Chin Wu[1†], En-Cheng Yang[1], Pei-Chi Wu[1], Po-Tse Lin[1] and Yueh-Lung Wu[1*]

[1] Department of Entomology, National Taiwan University, Taipei 106, Taiwan

Supplementary Table 1. RT-qPCR Ct values of immunity gene in bees. Analyses of mRNA were determined with quantitative RT-PCR and GAPDH was used as reference.

Repeat 1	Gene	Con	NaB	Imi	N/I	Gene	Con	NaB	Imi	N/I
**Immunity gene**	PHS	20.71520805	20.51193428	19.45803452	18.93142319	lys-2	20.91740799	20.71872139	19.90552521	19.65230942
	Toll	20.52795219	20.41496277	19.18340302	18.92816734	Lys-3	23.78959274	23.61431694	22.69478035	22.90368462
	SPZ	19.92673683	19.93584061	18.52032852	18.12283325	PGRPLC	19.81125069	19.84449196	18.91469383	18.52575493
	PGRPS2	19.48058128	18.9413929	17.98399734	17.40573502	Imd	20.7733345	20.91866493	19.9740963	19.86722565
	PGRPS3	20.2748642	19.90810966	18.93045616	18.45248032	Tak-1	20.10062218	20.81869888	19.34557343	18.91856384
	Myd88	19.92991447	19.93306351	18.59796333	18.27181625	Dredd	24.85956573	24.93122673	24.07640076	24.29953194
	Abaectin	21.75270653	21.91674232	20.5367794	20.28408241	Kenny	20.44877815	20.91369057	19.60560799	18.91347122
	Defensin-2	21.7816658	21.46515465	19.95273399	19.9275322	Tab	21.80714226	21.93795395	19.93737411	20.11269188
	Defensin-1	19.93415642	19.88124657	18.57893753	18.28642464	Hemipterous	23.60937119	23.92351723	23.30426979	23.93811417
	Cactus-1	19.77304268	21.93363953	18.99697113	20.01980209	basket	20.70324326	20.91261673	19.57631493	19.51320839
	Cactus-2	19.76769638	19.91484833	18.51890564	18.27122498	domeless	22.23427773	23.48724747	21.23065948	21.92170715
	Dorsal-1	21.31910324	22.7755661	19.93132973	21.345541	hopscotch	20.28547096	20.40277863	19.34047699	18.9205265
	PPOact	21.42386627	20.96088982	20.89534378	20.90087318	TEP7	20.01822853	20.19501877	19.23620415	18.92398643
	Hymenopt	25.914711	26.21421242	23.50039864	22.71022606	TEPA	19.85918808	19.92037964	18.76141167	18.47345543
	AmPPO	20.2844944	20.76877213	19.33384514	18.91472816	Relish	22.46938745	20.93383746	20.71383742	20.95029837
	PGRPS1	20.86989021	20.74970818	19.90823174	19.51489067	Dorsal-2	22.71393823	20.79837272	20.87837623	21.03039384
	Apidaec	26.37653923	25.96333313	23.92709541	24.17685127	GOX	24.73847562	21.87378224	21.81373664	21.59938724
	Apisimin	19.64513206	18.91958618	18.37586212	17.99814034	**GAPDH**	**21.91422272**	**21.94837189**	**21.74988174**	**21.91858864**
	Lys-1	20.37555504	20.27011871	19.18490791	18.92498398	**Actin**	**21.83745937**	**21.73827139**	**21.89352521**	**21.28390942**

Repeat 2	Gene	Con	NaB	Imi	N/I	Gene	Con	NaB	Imi	N/I
**Immunity gene**	PSH	21.90126801	19.91025162	20.01852989	20.1192627	lys-2	21.87983131	20.75523186	20.95179939	20.91619682
	Toll	21.73006248	19.91865921	19.73742485	19.91579247	Lys-3	25.82137489	25.40531731	25.59783363	25.90080643
	SPZ	22.20383263	19.91387749	19.88610077	19.89295006	PGRPLC	21.65472603	19.85390282	19.83091354	19.9125309
	PGRPS2	22.63985634	20.64828491	20.75939178	20.90307045	Imd	21.83875084	19.93214417	20.07106781	20.12182236
	PGRPS3	22.61207962	20.90472412	20.9190979	21.89390945	Tak-1	21.67453003	19.91010857	19.96138191	20.09889221
	Myd88	21.86929512	19.91026878	19.94975471	19.8571949	Dredd	26.39087868	25.98472977	25.86795044	25.91884804
	Abaectin	23.489254	21.91503906	21.90295029	21.89390945	Kenny	21.89116287	19.92218971	19.96461296	19.95959091
	Defensin-2	22.78177834	20.9272728	20.93264008	20.94418335	Tab	26.54448128	24.9146843	24.59867859	25.93694305
	Defensin-1	21.59089661	19.66390419	19.60807037	19.9114399	Hemipterous	21.92974472	20.08014679	19.99905396	20.26779366
	Cactus-1	24.99739265	22.93226433	22.97861862	23.44522858	basket	32.60590744	32.61072159	32.58789825	30.92504883
	Cactus-2	21.91947937	19.93005371	19.94178391	20.02757645	domeless	21.91835403	19.9771328	19.8645134	20.03670311
	Dorsal-1	32.3513298	30.56326485	29.91202927	31.1131916	hopscotch	21.76506424	19.97206688	19.91173363	19.97652817
	PPOact	21.91661072	19.89088821	19.7516861	20.03530121	TEP7	21.92674255	19.91826439	19.86409187	19.93877792
	Hymenopt	28.2917366	26.91680336	26.83589363	27.06047821	TEPA	22.6685524	20.84365654	20.94111443	20.92634201
	AmPPO	21.69142151	19.89927864	19.90284348	19.91510963	Relish	22.87215424	20.87821579	20.74247169	20.85637665
	PGRPS1	22.80894279	20.89601517	20.822752	20.86791611	Dorsal-2	22.6350174	20.84440613	20.89568138	20.91375542
	Apidaec	33.55959702	32.90567398	31.78964615	32.62903976	GOX	23.93930817	21.61336327	21.74194336	21.61144829
	Apisimin	21.90160179	20.32198334	20.33515358	20.94776535	**GAPDH**	**20.91230011**	**20.86617851**	**20.63577461**	**20.68984413**
	Lys-1	22.73223114	20.71954918	20.92889977	20.72332382	**Actin**	**21.3748455**	**21.34753154**	**20.93485766**	**20.93847564**

Repeat 3	Gene	Con	NaB	Imi	N/I	Gene	Con	NaB	Imi	N/I
**Immunity gene**	PSH	22.85195541	23.52850151	20.78788567	18.91007042	lys-2	22.75130463	22.42201614	21.60760498	19.91485596
	Toll	22.29002762	22.91770172	20.44158363	18.75269318	Lys-3	22.9088707	22.94047737	22.49440956	22.9501667
	SPZ	22.91444206	23.88239479	20.46306038	18.34092903	PGRPLC	22.90355682	23.57940483	20.81394196	18.83692551
	PGRPS2	22.82319069	23.89220238	20.39298439	18.11258698	Imd	21.91536713	21.71227264	20.47014427	18.89149284
	PGRPS3	22.79494667	22.91290665	20.79404068	18.83900452	Tak-1	22.46459198	22.91213036	20.46250153	18.87433243
	Myd88	22.76859856	23.6918869	20.83673668	18.90077591	Dredd	24.90350342	24.57098007	24.39987755	23.58740234
	Abaectin	23.9136219	23.73429298	22.58365059	20.95758438	Kenny	22.35942841	22.91645622	20.5305748	18.63877296
	Defensin-2	23.87520599	24.91870689	22.11698723	20.17152023	Tab	26.76384563	21.52938752	25.01339485	23.8429374
	Defensin-1	22.17343712	22.91070747	20.27535629	18.14273071	Hemipterous	21.1382637	20.6500721	19.90061188	17.93563652
	Cactus-1	23.92565727	24.44275856	22.89619637	21.22360802	basket	24.66680717	22.95161629	27.46933556	24.86878395
	Cactus-2	21.90714455	22.04024887	20.23747253	18.39697266	domeless	21.909338	21.91862488	19.58200836	17.49537086
	Dorsal-1	25.0492897	24.9233551	24.63524437	25.718153	hopscotch	22.85678673	23.52229691	20.75814056	18.79063988
	PPOact	22.0238455	23.91491699	21.92876244	19.44796753	TEP7	22.0123495	19.983938272	19.81221945	19.9894732
	Hymenopt	22.92457581	23.61570168	25.05664635	21.84347534	TEPA	22.90006256	22.665411	21.70206642	19.79315567
	AmPPO	23.39181709	24.58527756	20.92329597	18.4231987	Relish	22.91054153	23.33760262	20.77988243	18.91423416
	PGRPS1	22.74305916	23.4721241	20.57281303	18.89362526	Dorsal-2	22.37713432	23.93325424	20.47444153	18.3291378
	Apidaec	24.93005562	23.77373695	27.34207726	24.95049667	GOX	24.91569901	25.84288025	23.6265049	21.42064667
	Apisimin	23.61352921	25.24564552	21.38620949	18.92272949	**GAPDH**	**21.92043495**	**21.8896389**	**21.83766174**	**21.80863571**
	Lys-1	22.69757843	21.83793068	21.04	19.91400909	**Actin**	21.23556351	21.0029345	20.38495607	20.83974561

Supplementary Table 2. RT-qPCR Ct values of detoxification genes in bees. Analyses of mRNA were determined with quantitative RT-PCR and GAPDH was used as reference.

Repeat 1	Gene	Con	NaB	Imi	N/I
Detoxification genes	CYP9Q1	22.88910866	20.76655769	20.9164505	20.85453987
	CYP9Q2	22.97155952	20.90571022	20.93263245	21.07086754
	CYP9Q3	22.84084702	20.79967308	20.92991829	20.81857109
	CYP9S1	22.81266975	20.84011269	20.91563797	20.91378784
	CYP4G11	20.92723083	19.8495636	19.80864334	19.95101357
	CYP305D1	21.94115639	19.92511368	19.91328621	20.13895607
	GSTD1	22.48533058	20.50909233	20.91854286	20.75504112
	SODH2	22.62158775	20.6890316	20.78048706	20.92490578
	GSTD3	32.79460526	33.48877716	32.93555069	33.91448593
	Catalase	25.62077332	22.92051888	23.06710815	22.74554253
	Am2446	20.04862976	17.91112137	17.96661758	17.9142971
	CYP306A1	23.00805092	20.86812401	20.91759682	20.92892838
	PAKR1	21.63926697	19.94447899	19.90751839	19.91148567
	PAKC1	22.57203293	20.75882721	20.90512657	20.90830421
	CEst04	22.49116135	20.77426338	20.84901619	20.9145298
	CYP6AS14	21.63760948	19.79138374	19.91541481	19.88935852
	Am12900	14.99707031	13.06027699	12.80198288	12.91575432
	AmNOS	27.93827057	25.90815735	26.30570793	26.6773777
	**GAPDH**	**21.92043495**	**21.8896389**	**21.83766174**	**21.80863571**

Repeat 2	Gene	Con	NaB	Imi	N/I
**Detoxification genes**	CYP9Q1	21.24616051	20.62182808	20.91384888	19.75317764
	CYP9Q2	21.91709518	20.87528038	21.47477722	20.37530518
	CYP9Q3	22.92172813	22.00606537	22.15232658	20.35486031
	CYP9S1	22.93697166	23.0292511	21.93231583	19.67024231
	CYP4G11	17.94752502	16.76399422	16.97945213	16.30496025
	CYP305D1	22.67348289	24.28921509	20.86921692	18.8944416
	GSTD1	21.82103157	20.90431213	20.30720901	19.25928688
	SODH2	22.91212654	22.87837029	21.18533707	19.14382744
	GSTD3	30.64649963	31.24412346	28.91283607	30.24306297
	Catalase	23.43680954	23.60919189	21.91080666	20.41312027
	Am2446	19.79468346	18.0394856	18.92111206	17.13170242
	CYP306A1	24.97655869	25.46786118	23.91018486	21.49028397
	PAKR1	21.21821404	21.2848568	19.91243362	18.06672478
	PAKC1	21.8100071	21.80936432	21.17127419	19.90206528
	CEst04	22.9011364	23.02171516	21.55257988	19.8704071
	CYP6AS14	22.82089615	22.91546059	20.82303619	18.54348564
	Am12900	7.896350384	6.734662056	7.002850533	7.268334389
	AmNOS	26.85704994	26.92816353	25.60725975	24.29380417
	**GAPDH**	**20.8802166**	**20.91222191**	**20.87368774**	**20.85223579**

Repeat 3	Gene	Con	NaB	Imi	N/I
**Detoxification genes**	CYP9Q1	19.37551498	19.00841904	19.28723335	18.92700768
	CYP9Q2	20.29384041	20.93299866	20.2430687	19.78335762
	CYP9Q3	22.44496727	20.92437363	21.36010361	20.75983238
	CYP9S1	21.87207794	21.44946098	20.93353653	20.23694801
	CYP4G11	15.36518955	15.2002573	14.92164135	14.72861195
	CYP305D1	22.20919991	22.48836517	19.3141346	18.92869186
	GSTD1	20.08433723	19.67435837	19.4982338	18.91073036
	SODH2	22.34926987	20.75478745	19.91960907	19.78609085
	GSTD3	30.92148972	30.98521996	27.74403572	29.3501606
	Catalase	21.30845451	21.75979805	19.92307472	19.51907539
	Am2446	19.54732323	18.92012215	17.80229759	17.26478386
	CYP306A1	24.59558296	23.92348099	22.87581062	22.17892265
	PAKR1	20.21148872	19.91646004	18.77337646	18.42501259
	PAKC1	20.16337967	19.81370544	20.38997269	19.90581512
	CEst04	22.91851425	22.945364	20.85725403	20.4052906
	CYP6AS14	19.98187637	19.18362808	17.79489136	17.52120781
	Am12900	8.002841949	7.673294067	7.927249908	7.716110229
	AmNOS	25.94914436	25.16070175	24.46225548	23.91946793
	**GAPDH**	**18.68258858**	**18.91452408**	**19.56689072**	**19.89829826**
